# 

**Published:** 1882-05

**Authors:** 


### CONTENTS.

### *MISCELLANEOUS.*

PAGE.

The Obligations of the World to Hahnemann. E. Bayard, M. D., New York, . . . 201

Misrepresentations. Ad. Lippe, M. D., Philadelphia, . . . 205

Cimicifuga, in its Relation to Acute Catarrh. C. Carleton Smith, M. D., Philadelphia, . . . 211

The Centesimality of the Fincke High Potencies. B. Fincke, M. D., Brooklyn, N. Y., . . . 213

A Few Thoughts on the Study and Practice of Homœopathy. David Wilson, M. D., London, . . . 219

The Homœopathic Medical Society of Ohio, . . . 224

Hahnemann's Birthday; The Banquet of The Lippe Society. (Extract from Philadelphia *Bulletin.*) . . . 225

Notes on Genito-Urinary Therapeutics. E. J. L., . . . 228

Cough, with Vomiting. E. B. Nash, M. D., Cortland, N. Y., . . . 231

Fatal Errors. Ad. Lippe, M. D., Philadelphia, . . . 232

Index of Cough Symptoms, . . . 233

Letter from Dr. Fellger, . . . 233

Dr. Hering's Tables of Throat, Chest and Cough Symptoms.

### *CLINICAL BUREAU:*

A Plea for High Potencies. By the late Charles Julius Hempel, M. D., 234
Periscope, . . . 238
Notes and Notices, . . . 240

